# Impacts of service quality, brand image, and perceived value on outpatient’s loyalty to China’s private dental clinics with service satisfaction as a mediator

**DOI:** 10.1371/journal.pone.0269233

**Published:** 2022-06-08

**Authors:** Wenyi Lin, Wanxia Yin

**Affiliations:** 1 School of Public Administration, Jinan University, Guangzhou, China; 2 Common Prosperity and National Governance Institute, Jinan University, Guangzhou, China; Institute for Advanced Sustainability Studies, GERMANY

## Abstract

**Background:**

This study explores the effects and influence paths of service quality, brand image, perceived value, and service satisfaction on outpatients’ loyalty to China’s private dental clinics.

**Methods:**

A cross-sectional survey study was conducted in Dongguan City, Guangdong Province, China in January 2019. The participants were selected using the convenience sampling method. Of the 230 residents surveyed, 125 had received services in private dental clinics, being the valid sample of this study. A multiple linear regression model was used in exploring factors influencing patient loyalty. Subsequently, the path analysis was used in investigating the relationships among service quality, brand image, perceived value, patient satisfaction, and patient loyalty.

**Results:**

After the effects of demographic and socioeconomic variables were controlled, perceived value and patient satisfaction showed significant influences on patient loyalty. Path analysis indicated that perceived value, perceived quality, and expected quality have direct effects on patient satisfaction and have indirect effects on patient loyalty, and patient satisfaction is a mediator.

**Conclusion:**

Perceived service quality influences patient loyalty through the effect of patient satisfaction, which plays a key role in promoting patient loyalty. This study implies that managers in private dental clinics can gain support from customers by building customer loyalty toward dental clinics.

## Introduction

Patient loyalty, a behavioral intention or action to repurchase a preferred product or service consistently, is regarded as a key element of the business success of healthcare service providers [1–25]. In recent years, the Chinese government has gradually privatized healthcare services. Within this context, the private healthcare sector is growing rapidly, and the dental service industry has become a highly marketable business. The vigorous development of private dental clinics is conducive to solving the problems caused by the short supply of healthcare services in dental, general, and community hospitals. However, competition among private dental clinics is increasingly intensive [2, 3]. Hence, building patient loyalty toward dental clinics is quite important for dental service providers.

Most international empirical studies have confirmed the relationship between customer satisfaction and loyalty in the field of healthcare service [1, 4–6]. The literature about the impact of service quality on loyalty is extensive [7]. Some studies have discussed the relationships among service quality, service satisfaction, and service loyalty [8–11], whereas some analyzed the relationships among patient value, service satisfaction, and service loyalty [4, 5].

Most studies on patients’ loyalty to health care institutions in China have focused on public hospitals [1, 12–21]. The context of private institutions where patients pay for services is different from the context of public healthcare service institutions where most patients get services with medical subsidies. As such, patients are more concerned with service quality in private health institutions, and private health institutions pay more attention to patients’ loyalty.

Currently, only few studies in China have discussed private health service institutions [22–24]. A study considered the effect of perceived service quality on patient loyalty to private health service institutions. The results showed five perceived service quality, namely, accessibility, service attitude, medical quality, tangibility, and cost, which have a positive influence on inpatients’ loyalty to private hospitals [22]. Another study that collected data in *Chongqin* municipality showed that inpatients’ satisfaction plays a positive role in explaining their loyalty to hospitals [23]. A recent study conducted among 300 patients in 15 private hospitals in China indicated that service quality, patient perceived value, patient satisfaction are positively correlated with patient loyalty. Moreover, patient perceived value and patient satisfaction mediated the relationship between service quality and patient loyalty [24]. These three studies considered the impact of service quality and inpatients’ satisfaction on inpatients’ loyalty but did not consider the factors influencing outpatients’ loyalty and the influence path of these factors on outpatients’ loyalty. Considering that health services in China are undergoing privatization, this study explores the effects and influence paths of service quality, brand image, perceived value, and service satisfaction on outpatients’ loyalty to China’s private dental clinics. This study aims to make a contribution in the literature discussing trust-value-loyalty model (TVLM) [25] and customers in the private health service agencies. In addition, the findings of this study contribute to improving the outpatients’ loyalty in China’s private dental clinics.

## Methods

**Fig 1:**
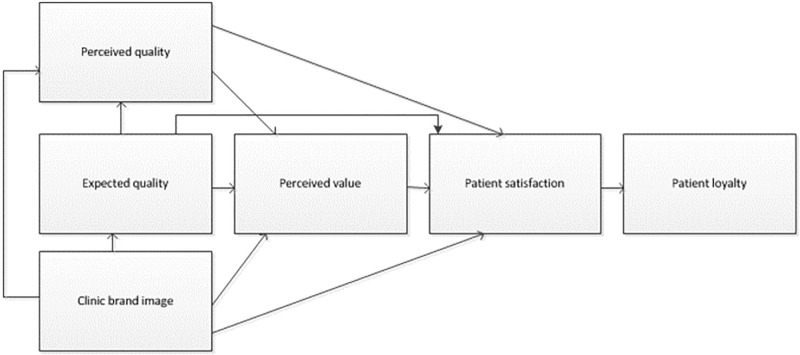
Theoretical framework.

The research framework of this study adopted the Chinese Customer Satisfaction Index (CCSI), which was proposed by Tsinghua University in 2000. It is currently a highly authoritative and representative model in China [12–19, 22, 23]. The CCSI constructs an integrated conceptual model comprising five determinants of patient loyalty, namely, perceived value, expected value, brand image, perceived value, and patient satisfaction (Fig 1).

On the basis of a research framework, two main hypotheses were constructed.

H1: Patient satisfaction has a positive influence on patient loyalty.
H2: Perceived quality, expected quality, clinic brand image, and perceived value have indirect effects on patient loyalty, and patient satisfaction has a mediating role.

As illustrated in Table 1 regarding the patient loyalty scale, brand image refers to the clinic’s recognition, credibility, and reputation. Perceived quality consists of 16 items, namely, physician medical technology, clinic medical equipment, patient treatment effect (degree of relief), handling complaints, waiting time for treatment, waiting time for payment, attitude of physician work, attitude of physician service, explanation of diagnosis and treatment, choice of treatment plan, health education, physician’s clothing, sanitary conditions, environmental comfort, facility sign, and convenience service (e.g., convenience of medical transportation and distance from residence). Expected quality includes overall impression, expectations of service items before treatment, expectations of treatment outcomes, and expectations of service effect. Perceived value comprises evaluations regarding service fees compared with the quality of services received and service quality compared with service fees paid by patients. Patient satisfaction is measured by overall satisfaction, satisfaction about services provided compared with expectations, and satisfaction about private clinics compared with other types of dental clinics.

**Table 1:** Patient loyalty scale.

Brand image	Clinic’s recognition
	Credibility
	Reputation
Perceived quality	Physician medical technology
	Clinic medical equipment
	Patient treatment effect (degree of relief)
	Handling complaints
	Waiting time for treatment
	Waiting time for payment
	Attitude of physician work
	Attitude of physician service
	Explanation of diagnosis and treatment
	Choice of treatment plan
	Health education
	Physician’s clothing
	Sanitary conditions
	Environmental comfort
	Facility sign
	Convenience service
Expected quality	Overall impression
	Expectations of service items before treatment
	Expectations of treatment outcomes
	Expectations of service effects
Perceived value	Evaluations regarding service fees compared with the quality of services received
	Service quality compared with service fees paid by patients
Patient satisfaction	Overall satisfaction
	Satisfaction about services provided compared with expectations
	Satisfaction about private clinics compared with other types of dental clinics
Patient loyalty	Willingness to choose private clinics next time
	Willingness to recommend private clinics to family members or friends

Patient loyalty includes two items: willingness to choose private clinics next time and willingness to recommend private clinics to family members or friends. Each item is coded as an ordinal variable on scales ranging from 1 to 5, and a high score indicates low satisfaction level. The Cronbach’s alpha values for perceived quality, expected quality, clinic brand image, perceived value, patient satisfaction, and patient loyalty range from 0.79 to 0.97, indicating that the internal consistency of these dimensions is good.

The present study is cross-sectional survey study and conducted in Dongguan City, Guangdong Province, China in January 2019. The rationale for selecting China’s private dental clinics as settings for data collection lies in the fact that dental service is the most privatized sector in China. Participants were selected with the convenience sampling method. A total of 230 residents were surveyed through an online platform in March, 2019. Among them, 125 had received services in private dental clinics, being the valid sample of this study. This study obtained ethical approval from School of Public Administration, Jinan University (201909). Online informed consent was obtained from each participant who had received services from private dental clinics. At the beginning of the questionnaire, the investigator explained the purpose of the research and the confidentiality of information, and the participants who were willing to fill in the questionnaire agreed to start filling out the questionnaire online.

Descriptive statistics were used in analyzing a sample’s characteristics. Then a multiple linear regression model was used in exploring the effects of service quality, brand image, perceived value, and patient satisfaction on patient loyalty. Unstandardized coefficient (B), standardized coefficient (Beta), and 95% confidence intervals (CIs) were reported, and the *P* values reported were two-tailed. The influences of service quality, brand image, perceived value, and patient satisfaction on patient loyalty were explored through path analysis. The maximum-likelihood estimation method was used in path analysis. The χ2 ⁄df, RMSEA, CFI, and SRMR indices were used in evaluating the fit of the analytical model. All data were analyzed with SPSS 24.0 and AMOS 21.0 (International Business Machines Corp: Beijing, China). Respondents with one missing variable were excluded from analysis.

## Results

Table 2 reports the characteristics of the respondents. Among the 125 respondents, 44 were men, accounting for 35.20% of the total sample. The majority of the participants were under 45 years old. The number of respondents who completed undergraduate or junior college was the highest, accounting for 64% of the total sample. Most participants (39.20% in total) had incomes of 1720 RMB and below.

**Table 2:** Sample description (n = 125).

	Mean or percent	Standard deviation	Minimum	Maximum
Patient loyalty	7.38	1.57	2	10
Patient satisfaction	11.08	2.05	3	15
Perceived value	7.14	1.43	4	10
Clinic brand image	10.98	1.99	3	15
Expected quality	14.92	2.43	8	20
Perceived quality	60.01	10.00	16	80
Gender(male)	35.20%			
Age				
25 years old and below	56.80%			
26–45 years old	21.60%			
46–60 years old	13.60%			
61 years old and above	8.00%			
Education				
Elementary school and below	9.60%			
Middle school	13.60%			
High school or vocational school	10.40%			
College	64.80%			
Graduate school	1.60%			
Income per month				
1720 RMB and below	39.20%			
1721–4000 RMB	28.80%			
4001–6000 RMB	16.80%			
6001–8000 RMB	10.40%			
8001 RMB and above	4.80%			

In 125 respondents, the mean of patient loyalty was 7.38 (SD = 1.57) out of 10. The average satisfaction score of all respondents was 11.08 (SD = 2.05) out of 15. The mean perceived value was 7.14 (SD = 1.43) out of 10. The mean of the clinic brand image was 10.98 (SD = 1.99) out of 15. The mean of expected quality was 14.92 (SD = 2.43) out of 20. The average score of perceived quality was 60.01 (SD = 10.00) out of 80.

The effects of perceived quality, expected quality, clinic brand image, perceived value, and patient satisfaction on patient loyalty are shown in Table 3. After the effects of demographic and socioeconomic variables were controlled, perceived value (Beta = 0.249; 95% CI: 0.118–0.380) and patient satisfaction (Beta = 0.372; 95% CI: 0.175–0.570) had positive influences on patient loyalty. By contrast, perceived quality, expected quality, and clinic brand image were not significantly associated with patient loyalty.

**Table 3:** Multiple linear regression model of patient loyalty in the private dental clinics (n = 125).

	B (95% CI*)	Beta (95% CI*)	P-value
Constant	-0.445 (-2.103–1.213)		0.596
Perceived quality	0.023 (-0.010–0.056)	0.148(-0.063–0.360)	0.168
Expected quality	0.006 (-0.104–0.116)	0.009(-0.161–0.180)	0.914
Clinic brand image	0.126 (-0.017–0.269)	0.160(-0.022–0.342)	0.084
Perceived value	0.274 (0.130–0.418)	0.249(0.118–0.380)	<0.001
Patient satisfaction	0.284 (0.134–0.435)	0.372(0.175–0.570)	<0.001
Gender	-0.120 (-0.470–0.230)	-0.037(-0.144–0.070)	0.497
Age	0.077 (-0.178–0.331)	0.048(-0.111–0.206)	0.553
Education	-0.074 (-0.308–0.160)	-0.050(-0.208–0.108)	0.533
Monthly income	0.076 (-0.062–0.214)	0.057(-0.047–0.162)	0.279

*CI- confidence interval.

The path influence of perceived quality, expected quality, brand image, perceived value, and patient satisfaction on patient loyalty were analyzed through path analysis, as shown in Table 4. Perceived value and patient satisfaction influenced patient loyalty significantly and directly. Perceived value, perceived quality, and expected quality had indirect effects on patient loyalty, and patient satisfaction was a mediator.

**Table 4:** The path coefficients in SEM (n = 125).

Variables		Variables	Estimate (Unstandarized)	Estimate (Standardized)	S.E.	C.R.	P
Expected quality	<---	Brand image	0.933	0.764	.071	13.174	<0.001
Perceived quality	<---	Expected quality	1.675	0.408	.345	4.859	<0.001
Perceived quality	<---	Brand image	2.213	0.441	.421	5.255	<0.001
Perceived value	<---	Brand image	0.053	0.074	.085	.625	.532
Perceived value	<---	Expected quality	-0.007	-0.012	.069	-.102	.919
Perceived value	<---	Perceived quality	0.084	0.589	.016	5.139	<0.001
Patient satisfaction	<---	Perceived value	0.196	0.136	.083	2.353	.019
Patient satisfaction	<---	Brand image	0.091	0.088	.079	1.156	.248
Patient satisfaction	<---	Perceived quality	0.113	0.551	.017	6.774	<0.001
Patient satisfaction	<---	Expected quality	0.160	0.189	.064	2.510	.012
Patient loyalty	<---	Patient satisfaction	0.465	0.440	.050	9.270	<0.001
Patient loyalty	<---	Perceived value	0.317	0.289	0.072	4.398	<0.001

Model fitness: χ2 ⁄df 4.687(P <0.05); RMSEA: 0.172 (90% CI: 0.089–0.268); CFI: 0.982; TLI: 0.909, SRMR: 0.000.

## Discussion

This study explored the effects and influence path of service quality, brand image, perceived value, and service satisfaction on outpatients’ loyalty in China’s private dental clinics. The findings indicated that adjusted demographic and socioeconomic variables, perceived value, and patient satisfaction have positive and direct influences on patient loyalty. Additionally, perceived value, perceived quality, and expected quality influenced patient loyalty by affecting patient satisfaction.

A positive impacts of outpatients’ perceived quality, perceived value, and service satisfactory on patients’ loyalty in public health institutions were explored in previous studies [1, 14, 17–19]. The overall image of a hospital and perceived quality in terms of technical service levels of doctors [13, 15, 17, 18], clinic’s facilities and environment [15, 19], waiting time [13], and doctor’s attitude [16, 17, 22] influence inpatients’ loyalty to doctors. Patient satisfaction is a mediator between perceived value and patient loyalty [12]. Our findings confirmed these relationships in private dental clinics. This study is the first time to examine CCSI model in private dental clinics in China.

This study is limited to private dental clinics, and the convenience sampling is applied to sample selection. Thus, the findings may not be generalized to all dental clinics due to selection bias. Moreover, the sample of this study cannot represent the population in China. Studies with larger samples are needed for increasing understanding of the relationships among service quality, satisfaction, and loyalty. Future studies can replicate this study design in other private dental clinics, to enhance the generalizability of the findings. Nevertheless, this study can help managers in private dental clinics in China in gaining support from customers through building customer loyalty toward dental services.

Furthermore, the findings of this study provide some practical implications. In practice, several dimensions associated with perceived service quality and perceived value should be improved in private clinics. Perceived service quality in this study included two items: waiting time for treatment and convenience service (e.g., convenience of medical transportation and distance from residence). The perceived value comprised the evaluations regarding service fees compared with the quality of services received and service quality compared with the service fee by patients.

The first concern is appointment service. Most private dental clinics do not have online appointment services in China, and thus potential patients cannot make appointments in advance and waiting time increases. When waiting time is extremely long, patients are less likely to receive services [15, 20, 26].

The second concern is the location of private dental clinics. Currently, high-quality large-scale private dental clinics are usually located in urban centers and are separated from residential communities, thereby reducing convenience. Popa and Daniela [27] and Crutzen et al. [28] claimed that online tools can provide convenient services for patients and can enhance patient’s loyalty to hospitals. On this basis, private dental clinics can fully use a *WeChat* public number or *WeChat* small program to provide a range of services, such as medical consultation, appointment registration, online diagnosis, fee inquiry, and online payment.

Another concern is service fee. When the investigators conducted the survey, most private dental clinics in Dongguan City do not have a clear price list and charging standards, which are generally in the form of fees for doctors’ oral quotations, doctors paying bills, and no billing information. This type of charging method may easily lead to random pricing and disorder within the dental clinics, which may reduce the satisfaction of perceived value.

To standardize the development of dental clinics, health authorities should strengthen the supervision of private dental clinics, and daily supervision and inspection of business qualifications, service quality, equipment safety, and price expenses should be strengthened. Moreover, examining and determining the qualifications of dentists are necessary [29].

## Supporting information

S1 File Questionnaire, Chinese version. (DOCX)

S2 File Questionnaire, English translation. (DOCX)

S3 File Data. (SAV)

## Abbreviations

CCSI: Chinese Customer Satisfaction Index

## References

[1] Zhou W.J., Wang Y.Y., Liu C.Y., et al. The Status and Influencing Factors of Patient Loyalty with Chronic Diseases. Chs Health Qual Manag 2017; 24: 63–66. [in Chinese].

[2] Zhu L.P., Zhou L., Chen Q., et al. Research on the status quo and development strategies of private hospitals in my country. Medicine and Philosophy (A), 2015, 36(10): 61 –64. [in Chinese].

[3] Yuan X.W. Analysis of constraints on the development of social capital-run medical institutions. Chinese Health Economics Economy, 2014, 33(09): 14–16. [in Chinese].

[4] Rahmani Z., Ranjbar M., Nadi G., Ali A. & Gorji M. The study of the relationship between value creation and customer loyalty with the role of trust moderation and customer satisfaction in Sari hospitals. *Elec*. *Phys*.2017; 9:4474–4478. 10.19082/4474 PMC5557124 28848619

[5] Miao R., Zhang H., Wu Q., Zhang J. & Jiang Z.H,B. Using structural equation modeling to analyze the patient value, satisfaction, and loyalty: a case study of healthcare in China, Int. J. Prod. Res.2019; 2:290–311.

[6] Nguyen NX, Tran K, Nguyen TA. Impact of Service Quality on In-Patients’ Satisfaction, Perceived Value, and Customer Loyalty: A Mixed-Methods Study from a Developing Country. Patient Prefer Adherence. 2021;15:2523–2538. Published 2021 Nov 17. doi: 10.2147/PPA.S333586 34819722 PMC8607125

[7] Ismail A., Yunan Y. Service quality as a predictor of customer satisfaction and customer loyalty. LogForum 2016;12: 269–283.

[8] Kondasani R., Panda R. Customer perceived service quality, satisfaction, and loyalty in Indian private healthcare. *Int J Health Care Qual Assur* 2015; 28: 452–467. doi: 10.1108/IJHCQA-01-2015-0008 26020428

[9] Fatima T., Malik S., Shabbir A. Hospital healthcare service quality, patient satisfaction and loyalty: An investigation in context of private healthcare systems. *Int*. *J*. *Qual*. *Reliab Manag*. 2018; 35:1195–1214.

[10] Ahmed S., Tarique Md. K., Arif I. Service quality, patient satisfaction and loyalty in the Bangladesh healthcare sector. *Int J Health Care Qual Assu* 2017;30: 477–488. doi: 10.1108/IJHCQA-01-2017-0004 28574325

[11] Meesala A., Justin P. Service quality, consumer satisfaction and loyalty in hospitals: Thinking for the future. *J Retail Consum Ser* 2018;40: 261–269.

[12] Feng ZH.Q., Duan X.M. An empirical study on the relationship among patient perceived value, satisfaction and patient loyalty. Econ. Manag 2008; 30: 170–176. [in Chinese].

[13] Cheng CH.Y., Guo Y.D., Zhong S.H. The loyalty relationship between outpatients and doctors. Mod. Prev. Med. 2009; 36: 47–50. [in Chinese].

[14] Xiao X. J. Research on Community Medical Service in Hubei Province Based on Customer Loyalty Model. *Mod Bus*. *Trade*. *ind*.2014; no.13: 68–70. [in Chinese].

[15] Zu X.L., Miao D.Q., Hu ZH.H. Analysis of Influencing Factors for Outpatient Loyalty to the General Hospital. Hosp Manage Forum 2014; 31: 15–17.[in Chinese].

[16] Zhang J., Chen T.B., Ni P. Study on the impact of inpatient service quality on patient satisfaction and loyalty. Chi J Health Stat 2016; 33: 684–686. [in Chinese].

[17] Wang C.H., Wang H., Li N.N., Zhao Y.W., Yin H.Y. Analysis of the influence of patients’ satisfaction degree on loyalty in the county public hospitals in Anhui Province. ACTA Univ. Medi Nanjing (So.c Sci.) 2017; 2: 134–137. [in Chinese].

[18] Song Q., Zuo X.W., Zhang T. Analysis of college students’ satisfaction and loyalty to school hospital service. Heilongjiang Sci 2018;9: 14–15 [in Chinese].

[19] Liu M.SH., Liu J., Zhang Y.N. Investigation and analysis of satisfaction, loyalty, and recognition of outpatients in third-level public hospitals in Guangdong Province. *Soft Sci*. *Health* 2019; 33: 78–81.[in Chinese].

[20] Liu S, Li G, Liu N, Hongwei W. The Impact of Patient Satisfaction on Patient Loyalty with the Mediating Effect of Patient Trust. Inquiry. 2021;58:469580211007221. doi: 10.1177/00469580211007221 33834860 PMC8040618

[21] Zhang L, Zhang Q, Li X, et al. The effect of patient perceived involvement on patient loyalty in primary care: The mediating role of patient satisfaction and the moderating role of the family doctor contract service. Int J Health Plann Manage. 2022;37(2):734–754. doi: 10.1002/hpm.3355 34697826

[22] Huang J., Chen F., Wu X.L., Tan H.W., Gong W.B., Lei X., et al. Inpatients’ loyalty in private hospital based on the perceived service quality. *Mod*. *Prev*. *Med*.,2017; 44: 90–94. [in Chinese].

[23] Huang J., Chen F., Wu X.L., Wang P., Sun Y., Lei X., et al. An empirical study on the relationship among patients’ experiences, satisfaction, and loyalty in private hospitals. *Chi Health Ser Manag* 2019;36:100–103. [in Chinese].

[24] Guo Y, Zhou Y, Xing X, Li X. Exploring the Relationship between Service Quality of Private Hospitals and Patient Loyalty from the Perspective of Health Service. Iran J Public Health. 2020;49(6):1097–1105.

[25] Gidaković Petar and Žabkar Vesna. “How industry and occupational stereotypes shape consumers’ trust, value and loyalty judgments concerning service brands.” Journal of Service Management 2021; 6:92–113.

[26] Bielen Frédéric & Demoulin Nathalie. Waiting time influence on the satisfaction-loyalty relationship in services. Managing Service Quality. 2007; 17. 174–193. doi: 10.1108/09604520710735182

[27] Popa A., Daniela V. Building patient loyalty using online tools. Annals of the University of Oradea: Econ Sci 2010; 19: 766–771.

[28] Crutzen R., Dianne C., de Vries Nanne K.. Bringing Loyalty to E-health: Theory Validation Using Three Internet-Delivered Interventions. *J Med Internet Res*. 2011; 13: e73. doi: 10.2196/jmir.1837 21946128 PMC3222180

[29] de Oliveira Bezerra, Ambrósio Lucas et al. “What determines patient loyalty in health services? An analysis to assist service quality management.” Total Quality Management & Business Excellence.2021; doi: 10.1080/14783363.2021.1960500

