# Supplementary material for: Impacts of service quality, brand image, and perceived value on outpatient’s loyalty to China’s private dental clinics with service satisfaction as a mediator
Source: PLoS One. 2022 Jun 8;17(6):e0269233. doi: 10.1371/journal.pone.0269233 (PMC9176788; doi:10.1371/journal.pone.0269233)
Supplement: S1 File — (DOCX) [file pone.0269233.s001.docx]

问卷编号：NO. 调查时间： 月 日

**东莞市民营口腔诊所就医者满意度调查**

亲爱的东莞市民：

您好！为进一步改善民营口腔诊所医疗服务的供给质量，营造良好的医疗服务环境，为卫生管理部门提供决策依据，现开展此项调查。感谢您在百忙之中参与调查，请您就个人在民营口腔诊所的就诊体验对问卷项目进行客观评价，为我们的课题研究提供真实可靠的参考数据。问卷填写大约需花费5分钟时间。本次调查采用匿名形式作答，会对您的所有信息保密，请放心填写。衷心感谢您的支持！

社会保障课题研究小组

**一、您的基本信息 （请在符合您情况的□前打“√”）**

1 您的性别是：

□男 □女

2 您的年龄是：

□25岁及以下 □26-45岁 □46-60岁 □61岁及以上

3 您的教育程度是：

□小学及以下 □初中 □高中或中专 □本科或大专 □硕士或硕士以上

4 您的职业是：

□公务人员（含事业单位职员） □企业职员 □商业或个体经营 □销售或服务人员

□专业技术人员（如会计师、律师、医护人员、记者等） □自由职业者 □工人

□农民 □离退休人员 □学生 □无职业 □其他，

5 您目前的平均月收入是：

□1720元及以下 □1721-4000元 □4001-6000元 □6001-8000元 □8001元及以上

6 请问您加入的医疗保险项目有（本题可多选）：

□公费医疗 □职工基本医疗保险 □城乡居民医疗保险

□商业医疗保险 □无参保 □其他，

7 请问您过往就诊于口腔医疗机构的原因主要是（本题可多选）：

□口腔疾病治疗 □口腔健康检查 □口腔美容 □口腔保健治疗（如洗牙）

□特殊时期的口腔保健（如孕期，儿童替牙期等） □从未就诊 □其他，

8 请问您是否曾在东莞市的民营口腔诊所就诊：

□是 □否（若您选择了“否”，问卷填答结束，感谢您的参与）

民营口腔诊所是指除了口腔专科医院、综合医院口腔科、社区医院口腔科等之外的个体私营口腔诊所。

**二、民营口腔诊所满意度调查（请您根据自身的就诊体验，在符合的选项下打钩“√”）**

**A 诊所形象**

| 序号 | 项目 | 非常  同意 | 比较  同意 | 一般,不确定 | 比较  不同意 | 非常  不同意 |
| --- | --- | --- | --- | --- | --- | --- |
| A9 | 他人曾向您推荐过民营口腔诊所 |  |  |  |  |  |
| A10 | 您认为民营口腔诊所的口碑信誉好 |  |  |  |  |  |
| A11 | 您认为民营口腔诊所的医资力量好 |  |  |  |  |  |

**B 信息一致性**

B12您对民营口腔诊所的了解程度是

□非常了解 □比较了解 □一般 □比较不了解 □非常不了解

B13在选择口腔医疗机构就诊时，您是否倾向于选择民营口腔诊所

□一定选择 □绝大数时候选择 □一般情况下选择 □少数时候选择 □几乎不选择

**C 顾客期望**

| 序号 | 项目 | 非常  同意 | 比较  同意 | 一般,不确定 | 比较  不同意 | 非常  不同意 |
| --- | --- | --- | --- | --- | --- | --- |
| C14 | 您对民营口腔诊所的总体印象好 |  |  |  |  |  |
| C15 | 就诊前，您认为民营口腔诊所可以很好地满足您的就医需求 |  |  |  |  |  |
| C16 | 就诊前，您对治疗结果的期望高 |  |  |  |  |  |
| C17 | 就诊前，您对医疗服务的期望高  （如医师技术水平、服务态度和就诊条件等） |  |  |  |  |  |

**D 顾客感知质量**（请您根据在民营口腔诊所的就诊体验做出满意度判断。）

| 序号 | 项目 | 非常  满意 | 比较  满意 | 一般,不确定 | 比较  不满意 | 非常  不满意 |
| --- | --- | --- | --- | --- | --- | --- |
| D18 | 医师医疗技术 |  |  |  |  |  |
| D19 | 诊所医疗设备 |  |  |  |  |  |
| D20 | 治疗效果（缓解程度） |  |  |  |  |  |
| D21 | 反映意见后诊所处理情况 |  |  |  |  |  |
| D22 | 就诊等候时间 |  |  |  |  |  |
| D23 | 付费等候时间 |  |  |  |  |  |
| D24 | 医师工作态度  （指询问病情、做检查和治疗时是否认真等） |  |  |  |  |  |
| D25 | 医师服务态度  （指是否尊重就医者、耐心对待就医者等） |  |  |  |  |  |
| D26 | 医师对病情诊断和治疗解释的详细程度 |  |  |  |  |  |
| D27 | 医师选择治疗方案时对患者意见的尊重程度 |  |  |  |  |  |
| D28 | 诊所内牙科卫生知识的宣传教育 |  |  |  |  |  |
| D29 | 医师衣着 |  |  |  |  |  |
| D30 | 卫生条件 |  |  |  |  |  |
| D31 | 环境舒适度 |  |  |  |  |  |
| D32 | 设施标志  如逃生通道、楼层指示牌等 |  |  |  |  |  |
| D33 | 便民服务  如就诊的交通便利程度、与住所的距离等 |  |  |  |  |  |

**E 顾客感知价值**

E34相对您在口腔诊所中接受的医疗服务质量水平，您觉得已支付费用

□非常值得 □比较值得 □一般 □比较不值得 □非常不值得

E35相对您已经支付的费用，您对服务质量水平的评价是

□非常满意 □比较满意 □一般 □比较不满意 □非常不满意

**F 顾客满意度**

F36您对民营口腔诊所的总体满意度

□非常满意 □比较满意 □一般 □比较不满意 □非常不满意

F37在民营口腔诊所的就诊过程中，您享受的医疗服务和您的期望比较

□非常满意 □比较满意 □一般 □比较不满意 □非常不满意

F38和其他类型的口腔医疗机构比较，您对民营口腔诊所的满意度评价是

其他口腔医疗机构主要包括：口腔专科医院、综合医院口腔科、社区医院口腔科等。

□非常满意 □比较满意 □一般，不确定 □比较不满意 □非常不满意

**G 顾客忠诚度**

G39您下次如果有口腔就诊需要，您愿意继续选择民营口腔诊所吗

□非常愿意 □比较愿意 □一般，不确定 □比较不愿意 □非常不愿意

G40您的家人或朋友如果有口腔就诊需要，您愿意向他们推荐民营口腔诊所吗

□非常愿意 □比较愿意 □一般，不确定 □比较不愿意 □非常不愿意

41.请您对东莞市民营口腔诊所的发展提出宝贵意见：

**问卷填答结束，再次感谢您的支持！祝您身体健康，生活愉快！**
