# Supplementary material for: Impacts of service quality, brand image, and perceived value on outpatient’s loyalty to China’s private dental clinics with service satisfaction as a mediator
Source: PLoS One. 2022 Jun 8;17(6):e0269233. doi: 10.1371/journal.pone.0269233 (PMC9176788; doi:10.1371/journal.pone.0269233)
Supplement: S2 File — (DOCX) [file pone.0269233.s002.docx]

Questionnaire number: No.

Survey time: month day

**Survey on satisfaction of patients in private dental clinics in *Dongguan***

Dear *Dongguan* citizens:

Hello! In order to further improve the supply quality of medical services in private dental clinics, create a good medical service environment, and provide decision-making basis for health management departments, this survey is being carried out. Thank you for taking part in the survey in spite of your busy schedule. Please objectively evaluate the questionnaire items on your personal experience in private dental clinics, and provide real and reliable reference data for our research. It takes about 5 minutes to fill out the questionnaire. This survey will be answered anonymously, and all your information will be kept confidential. Please feel free to fill it out. Thank you sincerely for your support!

Social security research group

**I. Your basic information (please tick "√" before □ which meets your situation)**

1 Your gender is:

□ Male □ Female

2 Your age is:

□25 years old and under □26-45 years old □46-60 years old □61 years old and above

3 Your education level is:

□ Primary school and below □ Junior high school □ High school or technical secondary school □ Undergraduate or junior college □ Master or above.

4 Your occupation is:

□ Civil servants (including employees of public institutions) □ Enterprise employees

□ Commercial or self-employed □ Sales or service personnel

□ Professional and technical personnel (such as accountants, lawyers, medical staff, journalists, etc.) □ Freelancer □ Workers

□ Farmers □ Retirees □ Students □ No occupation □ Other,

5 Your current average monthly income is:

□1720 *yuan* and below □1721-4000 *yuan* □4001-6000 *yuan* □6001-8000 *yuan* □8001 *yuan* and above

6 What medical insurance items do you join (multiple choices are allowed for this question):

□ Public medical care □ Basic medical insurance for employees □ Medical insurance for urban and rural residents □ Commercial medical insurance □ uninsured □ Other

7 What are the main reasons for your past visits to oral medical institutions (this question can be multiple choices):

□ Treatment of oral diseases □ Oral health examination □ Oral beauty □ Oral health care treatment (such as tooth cleaning)□ Oral health care in special period (such as pregnancy, dentition replacement for children, etc.) □ Never seen a doctor. □ Other,

8 Have you ever seen a doctor in a private dental clinic in *Dongguan*?

□ Yes □ No (if you choose "No", the questionnaire will be completed, thank you for your participation)

Private dental clinics refer to individual and private dental clinics except dental specialist hospitals, stomatology departments of general hospitals and community hospitals.

**II. Satisfaction Survey of Private Dental Clinics (please tick "√" under the eligible options according to your experience)**

**A clinic image**

| serial number | project | very  agree | compare  agree | General, uncertain | compare  disagree | very  disagree |
| --- | --- | --- | --- | --- | --- | --- |
| A9 | Others have recommended private dental clinics to you. |  |  |  |  |  |
| A10 | I think your private dental clinic has a good reputation. |  |  |  |  |  |
| A11 | Do you think that private dental clinics have good medical resources? |  |  |  |  |  |

**B information consistency**

B12 What is your knowledge of private dental clinics?

□ Very understanding. □ Know better. □ General □ Less understanding.

□ Very ignorant.

B13 When choosing dental medical institutions, do you prefer private dental clinics?

□ A certain choice □ Most of the time □ Usually □ A few times □ Almost no choice

**C customer expectation**

| serial number | project | very  agree | compare  agree | General, uncertain | compare  disagree | very  disagree |
| --- | --- | --- | --- | --- | --- | --- |
| C14 | Your general impression of private dental clinics is good. |  |  |  |  |  |
| C15 | Before seeing a doctor, do you think that private dental clinics can meet your medical needs well? |  |  |  |  |  |
| C16 | Before seeing a doctor, you had high expectations for the treatment results. |  |  |  |  |  |
| C17 | Before seeing a doctor, you had high expectations for medical services.  (such as doctors' technical level, service attitude and medical conditions, etc.) |  |  |  |  |  |

D Customer perceived quality (please judge your satisfaction according to your experience in private dental clinics. )

| serial number | project | very  be satisfied | compare  be satisfied | General, uncertain | compare  Dissatisfied | very  Dissatisfied |
| --- | --- | --- | --- | --- | --- | --- |
| D18 | Physician's medical technology |  |  |  |  |  |
| D19 | Clinic medical equipment |  |  |  |  |  |
| D20 | Therapeutic effect (remission degree) |  |  |  |  |  |
| D21 | After reflecting the opinions, the clinic handled the situation. |  |  |  |  |  |
| D22 | Waiting time for medical treatment |  |  |  |  |  |
| D23 | Paid waiting time |  |  |  |  |  |
| D24 | Physician's working attitude  (Refers to asking about the condition, whether you are serious when doing examination and treatment, etc.) |  |  |  |  |  |
| D25 | Physician's service attitude  (Refers to whether to respect and treat patients patiently, etc.) |  |  |  |  |  |
| D26 | The doctor's degree of detail in the diagnosis and treatment of the disease. |  |  |  |  |  |
| D27 | How much doctors respect patients' opinions when choosing treatment plans. |  |  |  |  |  |
| D28 | Publicity and education of dental hygiene knowledge in clinic |  |  |  |  |  |
| D29 | Physician clothing |  |  |  |  |  |
| D30 | sanitary condition |  |  |  |  |  |
| D31 | Environmental comfort |  |  |  |  |  |
| D32 | Facility sign  Such as escape routes, floor signs, etc |  |  |  |  |  |
| D33 | convenience service for the public  Such as the convenience of medical treatment, the distance from residence, etc. |  |  |  |  |  |

**E customer perceived value**

E34 Compared with the quality level of medical service you received in the dental clinic, do you think you have paid for it?

□ Very worthwhile. □ It is worth it. □ General □ It's not worth it. □ Very unworthy.

E35 Compared with the fees you have already paid, what is your evaluation of the service quality level?

□ Very satisfied. □ Satisfied. □ General □ Less satisfied. □ Very dissatisfied

**F customer satisfaction**

F36 Your overall satisfaction with private dental clinics

□ Very satisfied. □ Satisfied. □ General □ Less satisfied. □ Very dissatisfied

F37 Compare the medical services you enjoy with your expectations during the visit of private dental clinics.

□ Very satisfied. □ Satisfied. □ General □ Less satisfied. □ Very dissatisfied

F38 Compared with other types of dental medical institutions, what is your satisfaction rating of private dental clinics?

Other dental medical institutions mainly include: dental specialist hospital, dental department of general hospital, dental department of community hospital, etc.

□ Very satisfied. □ Satisfied. □ General, uncertain □ Less satisfied. □ Very dissatisfied

**G customer loyalty**

G39 If you need dental treatment next time, would you like to continue to choose private dental clinics?

□ Very willing. □ More willing □ General, uncertain □ Less willing □ Very reluctant

G40 If your family or friends need dental treatment, would you like to recommend private dental clinics to them?

□ Very willing. □ More willing □ General, uncertain □ Less willing □ Very reluctant

41. Please give your valuable suggestions on the development of private dental clinics in *Dongguan*:

**The questionnaire is finished. Thank you again for your support!**

**I wish you good health and a happy life!**
