# Supplementary material for: Impacts of service quality, brand image, and perceived value on outpatient’s loyalty to China’s private dental clinics with service satisfaction as a mediator
Source: PLoS One. 2022 Jun 8;17(6):e0269233. doi: 10.1371/journal.pone.0269233 (PMC9176788; doi:10.1371/journal.pone.0269233)
Supplement: Attachment — Submitted filename: responses to comments.doc [file pone.0269233.s004.doc]

PONE-D-21-24436

Impacts of service quality, brand image, and perceived value on outpatient’s loyalty to China’s private dental clinics with service satisfaction as a mediator

**Responses to Journal requirements:**

When submitting your revision, we need you to address these additional requirements.

1. Please ensure that your manuscript meets PLOS ONE's style requirements, including those for file naming. The PLOS ONE style templates can be found at

https://journals.plos.org/plosone/s/file?id=wjVg/PLOSOne_formatting_sample_main_body.pdf and

<https://journals.plos.org/plosone/s/file?id=ba62/PLOSOne_formatting_sample_title_authors_affiliations.pdf>.

Response: We have carefully read PLOS ONE's style requirements, and revised the manuscript’s format based on the requirements.

2. Please amend your current ethics statement to address the following concerns:

a) Did participants provide their written or verbal informed consent to participate in this study?

Response: Online informed consent was obtained from each participant who had received services from private dental clinics.

b) If consent was verbal, please explain i) why written consent was not obtained, ii) how you documented participant consent, and iii) whether the ethics committees/IRB approved this consent procedure.

Response: This study obtained ethical approval from School of Public Administration, Jinan University (201909). Online informed consent was obtained from each participant who had received services from private dental clinics. At the beginning of the questionnaire, the investigator explained the purpose of the research and the confidentiality of information, and the participants who were willing to fill in the questionnaire agreed to start filling out the questionnaire online.

3. Please include additional information regarding the survey or questionnaire used in the study and ensure that you have provided sufficient details that others could replicate the analyses. For instance, if you developed a questionnaire as part of this study and it is not under a copyright more restrictive than CC-BY, please include a copy, in both the original language and English, as Supporting Information.

Response: Yes. In the revised version, we have provided the questionnaire as part of this study. Please the supporting files.

4. We note that the grant information you provided in the ‘Funding Information’ and ‘Financial Disclosure’ sections do not match.

When you resubmit, please ensure that you provide the correct grant numbers for the awards you received for your study in the ‘Funding Information’ section.

Response: Thanks. We will make a revision when resubmitting the manuscript.

5. Thank you for stating the following in the Funding Section of your manuscript:

“The writing of this article was supported by Social Science Foundation of Guangdong Province（GD20CGL02）. The funders had no role in the design of the study, the collection, analysis and interpretation of data.”

We note that you have provided funding information that is not currently declared in your Funding Statement. However, funding information should not appear in the Acknowledgments section or other areas of your manuscript. We will only publish funding information present in the Funding Statement section of the online submission form.

Please remove any funding-related text from the manuscript and let us know how you would like to update your Funding Statement. Currently, your Funding Statement reads as follows:

“The writing of this article was supported by Social Science Foundation of Guangdong Province（GD20CGL02）. The funders had no role in the design of the study, the collection, analysis and interpretation of data.”

Please include your amended statements within your cover letter; we will change the online submission form on your behalf.

Response: Thanks. We have removed the funding information in the revised version and plan to provide the funding information in the Funding Statement section of the online submission form.

6. Please include your tables as part of your main manuscript and remove the individual files. Please note that supplementary tables (should remain/ be uploaded) as separate ""supporting information"" files.

Response: Thanks. We have inserted the figure and tables in the main manuscript.

Please review your reference list to ensure that it is complete and correct. If you have cited papers that have been retracted, please include the rationale for doing so in the manuscript text, or remove these references and replace them with relevant current references. Any changes to the reference list should be mentioned in the rebuttal letter that accompanies your revised manuscript. If you need to cite a retracted article, indicate the article’s retracted status in the References list and also include a citation and full reference for the retraction notice.
Response: Base on the reviewers’ suggestions, we have added the recent literature in the revised version. Concretely, the updating literature included:

6. Nguyen NX, Tran K, Nguyen TA. Impact of Service Quality on In-Patients' Satisfaction, Perceived Value, and Customer Loyalty: A Mixed-Methods Study from a Developing Country. Patient Prefer Adherence. 2021;15:2523-2538. Published 2021 Nov 17. doi:10.2147/PPA.S333586.

20. Liu S, Li G, Liu N, Hongwei W. The Impact of Patient Satisfaction on Patient Loyalty with the Mediating Effect of Patient Trust. Inquiry.

2021;58:469580211007221. doi:10.1177/00469580211007221.

21. Zhang L, Zhang Q, Li X, et al. The effect of patient perceived involvement on patient loyalty in primary care: The mediating role of patient satisfaction and the moderating role of the family doctor contract service. Int J Health Plann Manage. 2022;37(2):734-754. doi:10.1002/hpm.3355.

24. Guo Y, Zhou Y, Xing X, Li X. Exploring the Relationship between Service Quality of Private Hospitals and Patient Loyalty from the Perspective of Health Service. Iran J Public Health. 2020;49(6):1097-1105.

25. Gidaković, Petar and Vesna Žabkar. “How industry and occupational stereotypes shape consumers' trust, value and loyalty judgments concerning service brands.” Journal of Service Management 2021; 6:92-113.

28. Bielen, Frédéric & Demoulin, Nathalie. Waiting time influence on the satisfaction-loyalty relationship in services. Managing Service Quality. 2007; 17. 174-193. 10.1108/09604520710735182.

29. Bezerra de Oliveira, Lucas Ambrósio et al. “What determines patient loyalty in health services? An analysis to assist service quality management.” Total Quality Management & Business Excellence.2021; DOI: 10.1080/14783363.2021.1960500.

**Responses to Reviewers' comments:**

Reviewer's Responses to Questions

Reviewer #1: Thank you for providing me the opportunity to read an interesting article. Although the idea is unique and exciting, a few areas need to improve before publication. The contributions of the article need more emphasis. The literature regarding the study is not up to date. The authors are advised to add some recent literature from 2021 and 2022. Good Luck.

Response: Thanks. We have stated contributions of the article more clearly in the revised manuscript, please see Page 3 Lines11-14:” This study aims to make a contribution in the literature discussing trust-value-loyalty model（TVLM）[25] and customers in the private health service agencies. In addition, the findings of this study contribute to improving the outpatients’ loyalty in China’s private dental clinics.” . And we have provided updated literature in the revised manuscript, please see Page 3 Line1-5: “A recent study conducted among 300 patients in 15 private hospitals in China indicated that service quality, patient perceived value, patient satisfaction are positively correlated with patient loyalty. Moreover, patient perceived value and patient satisfaction mediated the relationship between service quality and patient loyalty [24]. “Page 3 Line11-12 “T This study aims to make a contribution in the literature discussing trust-value-loyalty model（TVLM）[25]”. We have searched the latest articles published on the journals indexed in the web of science in theses two years, but the articles related to the customers’ loyalty in the private health service agencies are scarce.

Reviewer #2: I am glad to review and assess this exciting article, entitled " Impacts of service quality, brand image, and perceived value on outpatient’s loyalty to China’s private dental clinics with service satisfaction as a mediator" . I am fully satisfied and accept this manuscript for publication

Reviewer #3: Great work. It makes a very insightful reading . The analysis are very well done and the recommendation is apt. A good demonstration of the principles of research. Looking forward for more research and update with same field .

Reviewer #4: This research focuses on the impacts and influence paths of service quality, perceived value, brand image, and service satisfaction on outpatients’ loyalty to China’s private dental clinics.
This paper's contention is built on a suitable base of hypotheses, concepts, or other thoughts and is also well designed with appropriate methods.

Findings displayed clearly and examined suitably

This paper identify clearly between any implications for research but need to mention details about theoretical implications and Practical implications. This paper have bridge the gap between theory and practice.
Response: Thanks. We have stated more theoretical contributions and Practical implications on Page 3 Lines 11-14 :” This study aims to make a contribution in the literature discussing trust-value-loyalty model（TVLM）[25] and customers in the private health service agencies. In addition, the findings of this study contribute to improving the outpatients’ loyalty in China’s private dental clinics.”

The authors need to recheck the clarity of expression and readability, such as sentence structure.
Response: We have asked native speaker to proofread the revised manuscript, and tried our best to make the language more readable.

1.: Need to cite more references from the previous study.

Response: Thanks. We have cited more references from the previous studies, please see references:

6. Nguyen NX, Tran K, Nguyen TA. Impact of Service Quality on In-Patients' Satisfaction, Perceived Value, and Customer Loyalty: A Mixed-Methods Study from a Developing Country. Patient Prefer Adherence. 2021;15:2523-2538. Published 2021 Nov 17. doi:10.2147/PPA.S333586.

20. Liu S, Li G, Liu N, Hongwei W. The Impact of Patient Satisfaction on Patient Loyalty with the Mediating Effect of Patient Trust. Inquiry.

2021;58:469580211007221. doi:10.1177/00469580211007221.

21. Zhang L, Zhang Q, Li X, et al. The effect of patient perceived involvement on patient loyalty in primary care: The mediating role of patient satisfaction and the moderating role of the family doctor contract service. Int J Health Plann Manage. 2022;37(2):734-754. doi:10.1002/hpm.3355.

24. Guo Y, Zhou Y, Xing X, Li X. Exploring the Relationship between Service Quality of Private Hospitals and Patient Loyalty from the Perspective of Health Service. Iran J Public Health. 2020;49(6):1097-1105.

25. Gidaković, Petar and Vesna Žabkar. “How industry and occupational stereotypes shape consumers' trust, value and loyalty judgments concerning service brands.” Journal of Service Management 2021; 6:92-113.

28. Bielen, Frédéric & Demoulin, Nathalie. Waiting time influence on the satisfaction-loyalty relationship in services. Managing Service Quality. 2007; 17. 174-193. 10.1108/09604520710735182.

29. Bezerra de Oliveira, Lucas Ambrósio et al. “What determines patient loyalty in health services? An analysis to assist service quality management.” Total Quality Management & Business Excellence.2021; DOI: 10.1080/14783363.2021.1960500.

2. Contributions of the paper is convincing.

3. page 9, second paragraph need more citation.

Response: Thanks. We have cited more references from the previous studies, please see Page 10 Line12 “[15,20,26]”.

4. It requests professional editing so that readers' coherence can be improved.

Response: We have asked native speaker to proofread the revised manuscript, and tried our best to make the language more readable.

Reviewer #5: In China, where private health care services are very expansive, the study with this framework is interesting. Overall, this study is presented well. It would be good to work more on the limitation section.

Response: Thanks. We have stated more limitations on Page9,Lines 20-27 and Page 10, Line 1: “This study is limited to private dental clinics, and the convenience sampling is applied to sample selection. Thus, the findings may not be generalized to all dental clinics due to selection bias. Moreover, the sample of this study cannot represent the population in China. Studies with larger samples are needed for increasing understanding of the relationships among service quality, satisfaction, and loyalty. Future studies can replicate this study design in other private dental clinics, to enhance the generalizability of the findings. Nevertheless, this study can help managers in private dental clinics in China in gaining support from customers through building customer loyalty toward dental services. ”
